# Impact on Hearing Due to Prolonged Use of Audio Devices: A Literature Review

**DOI:** 10.7759/cureus.31425

**Published:** 2022-11-12

**Authors:** Shruti S Dehankar, Sagar S Gaurkar

**Affiliations:** 1 Otolaryngology - Head and Neck Surgery and Surgical Oncology, Jawaharlal Nehru Medical College, Datta Meghe Institute of Medical Sciences, Wardha, IND

**Keywords:** audiometry, audio devices, hearing impairment, headphones, nihl

## Abstract

Perpetuated exposure to higher decibels is one of the leading causes of noise-induced hearing loss (NIHL). The occurrence of NIHL has likewise been expanding in kids and adolescents. NIHL in youngsters and adolescents is connected with personal music players, which need earphones or headphones, and have powerful sound tones. The effects of such leisure activities and their relation to NIHL remains poorly studied. Various studies have been conducted to establish the degree of correlation between audio device usage and hearing disablement; recent studies show more agreeable data about the same. Therefore, the use of earphones and their effects on hearing needs to be well assessed, considering the high prevalence of the problem and the relative lack of literature for the same.

The intent of the following review is to gather information regarding the manifestations of impairment in hearing due to audio devices with the objective to assess the correlation between hearing impairment and the use of audio devices. Also, guidelines established by Central Pollution Control Board regarding noise pollution need to be revised as per the present-day setting. The implementation of these guidelines is the responsibility of the companies that design and produce different types of audio devices. These devices should be made ear-safe according to standard safety guidelines related to hearing impairment from loud noise. This review has taken into consideration 23 authentic studies from all around the globe that state a positive or negative result after studying the correlation between NIHL and long-term noise exposure. The findings of this narrative review indicate that leisure activities involving the use of audio devices pose a risk of NIHL, and a need for further research in this domain is seen, given the ubiquitous and ever-increasing nature of their consumption.

## Introduction and background

Noise-induced hearing loss (NIHL) is an impairment in hearing due to perpetuated exposure to loud sounds. This is classified as sensorineural hearing loss, which usually presents with bilateral, irreversible, and progresses with continual exposure to higher decibels. It is a general medical condition that can restrict regular day-to-day activities. The threat of disablement in hearing because of prolonged exposure to noise is present in about 12% of the population worldwide, which is also among the most preventable causes [1]. NIHL was originally identified as an occupation-based disease, and the World Health Organization assessed that 33% of all instances of hearing misfortune could be credited to clamour openness [1-3].

The Global Burden of Disease Study [4] found that hearing loss is the fourth leading cause of disability globally. The primary effect of hearing loss is impaired communication, which adversely affects relationships with family and friends and creates difficulties in the workplace. Untreated hearing loss in adults also has indirect health, psychosocial, and economic effects leading to social isolation and reduced quality of life [5,6]. Additionally, hearing disablement has financial repercussions, including higher healthcare expenditures, costs associated with the special schooling requirements of children who have deafness, and lower job performance that results in individual income losses [7]. The occurrence of NIHL has likewise been expanding in kids and adolescents. About 40% of understudies between the ages of 16 and 25 years and 1% of youngsters attending class were accounted for to have side effects of NIHL in 1996. All the more, as of late, 12.5% of understudies between the ages of six to 19 years were detected by doctors to have noise-induced threshold shifts [5,8]. NIHL in youngsters and adolescents is connected with commotions created during sporting and relaxation exercises. Noisy toys, band practices, engine sports, and the loud clamour in theatre lobbies and nightclubs have the potential to negatively impact hearing [6,8]. The utilisation of individual music players can likewise cause commotion prompted hearing misfortune in adolescents [9]. Personal music players, which are connected to earphones or headphones, have powerful sound tones, and their maximum volume level can reach 78-136 dB [10]. Music and NIHL are one of the most preventable health issues among young people. Currently, manufacturers are not taking on the responsibility of educating the public about the hazards of careless use of their products [11]. In some cases, NIHL may lead to permanent damage, and a hearing aid or an implant of the middle or inner ear is unavoidable [12].

## Review

Studies carried out in the period between 1985-1990

A pilot study was made by Lees et al. in 1985 to assess the prevalence of high-frequency hearing loss due to non-occupational noise exposure in 60 randomly selected students. The audiometric assessment was performed on 30 males and 30 females between the ages of 16-25 years. Using a 10 dB attenuation of hearing at one test frequency as a positive result, 40% of the subjects had evidence of noise-induced hearing loss. The majority of positive cases manifest hearing impairment at 6000 Hz and not the classically accepted 4000 Hz frequency. A highly significant correlation was found between leisure noise exposure and positive test results [13].

Lee et al. in 1985 conducted another study. Sixteen individuals participated in this pilot study in which they listened to earphones at their habitual maximum volume for three hours. A total of seven participants showed notable transient shifts, which returned to normal within a day. Above mentioned facts indicated that recreational sound often caused transient shifts, and rules for auditory public sustainability must be laid down [14].

In 1985, Catalano et al. carried out a study involving three well-known testing models: Bruel and Kjaer sound level meter, Octave band filter, and Artificial ear. Researchers provided around 200 students with a questionnaire, and audiometric testing (frequencies used: 250-8000) was carried out. Nearly 31% of all radio users surpassed the maximum permissible noise dose considering the Occupational Safety and Health Administration (OSHA) criteria. Fifty per cent of the "at risk" group went above and beyond the risk threshold [15].

In 1990, about 500 youngsters (15-24 years) in Hong Kong were interviewed by Wong et al. about their audio device usage frequency. More than 80% of them reported using audio devices for the last three years averaging about four and a half hours per week. The audiometric and otoscopic examination did not suggest a strong correlation between personal cassette player (PCP) users and NIHL, as most of them used these devices at safe levels [16].

Studies carried out in the period between 1991-1995

Hetu and Fortin, in 1995, took into consideration highly amplified music as a potential risk of hearing damage. Intense pulsations (rate 2 Hz), a limited dynamic range, and a sloping spectrum with the maximal energy in the 1/3-octave centred at 63 Hz were found to be the characteristics of party music. Persistent threshold shift seemed extremely unlikely for people who routinely listened to loud music, even though temporary threshold shift is anticipated for various durations of exposure to this type of sound [17].

Studies carried out in the period between 1996-2000

In 1999, according to Zenner's analysis of clinical statistics, it was found that one in ten teenagers suffered from NIHL caused by leisure-time noise [18].

Studies carried out in the period between 2001-2005

In 2002, Mazlan et al. conducted an analysis on 136 Client Service Representatives from a Malaysian telecommunication company who utilised audio headsets during their office hours. The objective of this study was to quantify the frequency of auditory canal infection and numerous ear, nose, and throat infections connected to it. Using the Amplaid 309 Clinical Audiometer, their hearing limits also were determined. In the subjects, we did not discover any common disease of the external ear canal. In four cases, the centre ear condition persisted, and in four patients, the wax was impacted. Twenty-five participants were examined for hearing impedance (21.2 per cent). However, because the use of earphones did not indicatively affect the high frequencies, there was little correlation between hearing disability and sound exposure [19].

Studies carried out in the period between 2006-2010

In a study conducted in China by Peng et al. in 2007, hearing impairment was discovered in 14% of the ears of 120 youngsters who used personal listening devices (PLD). These 120 PLD users, alongside 30 youngsters with normal hearing, were subjected to audiometry of both 'conventional' and 'extended high-frequency' types. The obtained results indicated that prolonged use of PLD contributed to hearing damage. The research also demonstrates that extended high-frequency audiometry is a reliable technique for identifying hearing loss brought on by noise at the initial stages [20].

In an article published in 2007, Vogel et al. carried out a qualitative survey of focus-group discussions with adolescents aged 12 to 18 years from two large secondary schools (one urban and one rural) for pre-vocational and pre-university education. The semi-structured question route was theoretically drafted keeping in mind the protection motivation theory. The majority of young people revealed that they frequently listened to their MP3 players at the highest possible volume, particularly male students and students from pre-professional schools. Most young people claimed that they would not identify any limitations on their propensities for being open to music. They came to the conclusion that interventions should concentrate on educating young people about loud music's potential dangers and how to protect themselves, with a particular focus on pre-professional school students. There may be a need for MP3 player volume-level guidelines in addition to customised MP3 player modifications and hearing instruction for children [21].

Vogel et al., in 2009, focused on 1687 young people (12-to 19-year old). Of all members, 90% detailed paying attention to music via headphones on portable audio devices; about 29% were arranged as audience members in danger of hearing misfortune because of assessed openness of 89 dBA for more than one hour out of every day. Compared with listeners not at risk for hearing loss, at-risk listeners were more likely not to live with both guardians, to experience satisfaction of listening to higher decibels, to give an account of increased propensity strength connected with risky MP3 listening, and were not much inclined to be roused to safeguard their ears. The most reliable indicator of high-risk listening behaviour was propensity strength, suggesting that it might be challenging to change the young generation's intentional behaviour and that a variety of different approaches might be necessary to prevent music-related hearing impairment [22,23].

In 2009, Mostafapour et al. conducted a potential ear testing among a group of students aged above 18 years who presented with a history of exposure to various sources of higher decibels of leisure noise. Volunteered students were subjected to typical auditory examinations. In the study, it was identified that the presence or absence of particular notches in audiograms could not be linked to a particular noise-pollution source, and a low degree of correlation was found between PLD users and NIHL. But this could not indicate a total disregard for the correlation between NIHL and prolonged use of audio devices in larger, more exposed populations [24].

In 2010, McNeill et al. evaluated the possible threat to hearing incidental to the usage of handy audio devices. A 49-item survey measuring client listening preferences and emotional aspects of hearing wellness was completed by 28 college freshmen (12 men and 16 women, ages 17 to 23). In a variety of study halls with foundation noise levels between about 40 and 55 dBA, sound level estimations of members' self-recognised common and "considering the worst possible scenario" volume levels were made. The term of usage was about two hours each day for six and a half days. The interquartile ranges (IQR) at average and 'worst case' volume settings were IQR=12 and IQR=9, respectively. Nineteen students who participated in the survey said they occasionally experienced a potential disturbance's adverse side effects, which led to hearing loss. Between those who had experienced tinnitus and those who had not, there were stark differences in MP3 client listening styles [25]. Table 1 mentions all the studies from 1985 to 2010 showing a positive association between NIHL and audio device usage.

**Table 1:** Studies showing a significant positive correlation between audio device usage and NIHL (1985-2010). NIHL = noise-induced hearing loss

Sr. No.	Year of Study	Author
1	1985 (pilot study)	Lee et al. [14]
2	1985	Catalano et al. [15]
3	1999	Zenner et al. [18]
4	2007	Peng et al. [20]
5	2009	Vogel et al. [22,23]
6	2010	McNeill et al. [25]

Studies carried out in the period between 2011-2015

In 2011, Schlauch and Carney conducted a study to calculate the percentage of false-positive results for guidelines proposed to detect early NIHL by studying audiograms for typical NIHL-associated notches. Audiograms of children belonging to the age group of 6-11 years showed no significant findings. Inadmissible high false-positive values were obtained after applying the pass-fail criteria to the data collected for measuring hearing acuity. Getting rid of systematic calibration errors, repeating and averaging threshold measurements, and utilising headsets with lower variability for 6.0 and 8.0 kHz, two frequencies vital for distinguishing noise notches, all have the potential to increase audiometric precision [26].

Another study in 2011, conducted by Punch et al., was aimed at assessing PLDs and their impact on hearing. It was found that young adults and teenagers seem to view the dangers of PLD use to ears somewhat differently. Therefore, messages intended to propose actions they may take to avoid or minimise these dangers must be targeted in order to have the best results [27].

In a cross-sectional study in November 2011 by Ansari et al., a 20-item questionnaire based on headphone usage was given to 2,400 random high school attendees. The results displayed that about 45% of the involved population had a record of hearing issues (the percentage of females was notably more than that of males). About 37% of the respondents confessed to listening to music non-stop, and about 50% stated that volume was kept at higher than usual levels. This included use of various kinds of audio devices, and only about 16% of students were not using any kind of music device. This study observed that the depicted hearing habits of students were pretty risky [28].

A 2013 article by Skånland investigated the use of music players and their role as affect regulators. This article stated that music, if integrated into one's life on a daily basis, would help us to regulate our emotions and achieve desirable outcomes in most situations. With newer portable technology such as MP3 players, it becomes easier to access music. This is an excellent tactic, as any hearing damage caused would be insignificant when compared to detrimental side effects of other risky regulation tactics such as alcoholism and smoking [29].

In 2013 itself, Sulaiman et al. assessed early hearing impacts connected with PLD utilisation in 35 youthful grown-up PLD clients and their age-and sex-matched controls using a mix of typical and extended high-frequency audiometry along with Transient-Evoked Otoacoustic Emission (TEOAE) and Distortion Product of Otoacoustic Emission (DPOAE) estimations. Increased values of the mean hearing threshold at prolonged high frequencies in PLD clients, along with decreased amplitudes of TEOAE and DPOAE, were observed, which could be inferred as a leading road to hearing impairment [30].

In 2014, Naik et al. conducted a study amongst 1000 students from a reported institute. Four groups were made, Group A involving 250 understudies who had a propensity for listening to music using earphones for no less than two hours daily, Group B including the same number of understudies who listen to headphones for under one hour each day and Group C including 250 understudies who once in a while use headphones but do use speakers often and lastly Group D involving the rest 250 understudies who are not accustomed to listening music via headphones. All four groups were exposed to pure tone audiometry. About 5%- 10% of understudies from Group A and 1%- 5% from Group B depicted high-frequency hearing loss. No significant hearing disablement was observed in Group C and D. This suggested a strong correlation between the duration of hearing and NIHL [31].

In 2015, Keppler et al. evaluated the effects of leisure-noise exposure on hearing function in about 160 young adults. Weekly and lifetime equivalent noise exposure calculations were the basis of the division of participants into low, intermediate, and highly exposed categories. Between the three groups, there were no appreciable changes in hearing thresholds, TEOAE amplitudes, or DPOAE amplitudes. More than 85% of subjects complained of temporary post-exposure tinnitus. No significant difference between the participants of the three groups was appreciated in the short duration [32].

Studies carried out in the period between 2016-2020

In 2019, Zia et al. directed a detailed observational review at the Outpatient Department of a renowned hospital in Karachi, Pakistan. Data in regards to the earphone, use of Q-tips and swimming and their manifestations as ear disease were studied. Injury to the auditory canal, tingling sensations, ear discharge, tinnitus, and hearing disablement were some of the occurrences as per the data gathered through a pre-organised survey. About 56% of the population under consideration used headphones, and a majority of them presented with infection of the ear, itching, and hearing damage [33].

Byeon, in 2020, led an observational review. The study was based on South Korean youths who were habitual of using headphones in public places with a loud environment. This study assessed around 530 subjects (12-19 years) who also were a part of a national survey in the past. Teenagers who were presented to high commotion levels through earphones in an uproarious climate, along with teenagers whose headphone utility extended 80 mins each day, had about 20%-25% predominance of possible hearing loss over others. This indicated that these students were 4.5 times more prone to hearing damage than others. The researcher noticed that longitudinal examinations are expected to give proof of causality between headphone use and hearing loss [34].

Studies carried out post-2020

In a 2021 review by Pienkowsk, the researchers address modern-day fun, which includes party clubs, sports stadiums and concerts, as "leisure noise". NIHL is one the most common preventable manifestations of this leisure noise, tinnitus and hyperacusis being the others. A much more comprehensive range of settings should implement noise limitations, and public health initiatives should place more emphasis on promoting aural conservation through education [35].

In 2022, AlQahtani et al. played out a cross-sectional review to concentrate on the mindfulness of NIHL and hearing misfortune. The goal of the evaluation was to determine how much awareness there is regarding the connection between using headphones and NIHL. The poll included about 40 questions, which were divided into as many as six categories. The review incorporated a total of 1086 members. Inspected populace's age went from 18 to 55 years. Hearing issues were persistent in people who were exposed to noisy work environments and were considerably greater when listening sessions lasted more than five hours per day. The age of the subject, loudness levels he/she is exposed to, and weekly session length were some of the intangibles taken into consideration. A low degree of public knowledge about hearing loss due to bad hearing habits was reported [36]. Table 2 tabulates studies carried out after 2010 with positive results for impacted hearing due to the usage of audio devices.

**Table 2:** Studies showing a significant positive correlation between audio device usage and NIHL (2011 onwards). NIHL = noise-induced hearing loss

Sr. No.	Year of Study	Author
1	2011	Ansari et al. [28]
2	2013	Sulaiman et al. [30]
3	2014	Naik et al. [31]
4	2019	Zia et al. [33]
5	2020	Byeon et al. [34]
6	2022	AlQahtani et al. [36]

## Conclusions

The term NIHL literally means any degree of hearing disablement caused due to prolonged noise exposure. With the advent of smartphones and various applications such as YouTube and other social media sites and video streaming platforms, leisure time activity and usage of earphones, headphones have become almost ubiquitous amongst the youth of developed and developing nations. Not only that, but high-quality headphones are also used during gaming, which involves the simulation of loud and sudden noises, such as those of gunshots, blasts, screams, and car crashes, delivered to the ear at close proximity, which are usually reasons for premature hearing loss.

Therefore, the use of earphones and their effects on hearing needs to be assessed, given the high prevalence of the problem and the relative paucity of literature on the same. After taking into consideration 23 authentic studies regarding the correlation between NIHL and sound exposure, the findings of this narrative review indicate that leisure activities involving the use of audio devices pose a threat of NIHL, and further research is needed in this particular area, given the ubiquitous and ever-increasing nature of their consumption.
